# A Neural Network Approach to Intention Modeling for User-Adapted Conversational Agents

**DOI:** 10.1155/2016/8402127

**Published:** 2015-12-27

**Authors:** David Griol, Zoraida Callejas

**Affiliations:** ^1^Department of Computer Science, Carlos III University of Madrid, Avenida de la Universidad 30, 28911 Leganés, Spain; ^2^Department of Languages and Computer Systems, University of Granada, CITIC-UGR, C/ Pdta. Daniel Saucedo Aranda s/n, 18071 Granada, Spain

## Abstract

Spoken dialogue systems have been proposed to enable a more natural and intuitive interaction with the environment and human-computer interfaces. In this contribution, we present a framework based on neural networks that allows modeling of the user's intention during the dialogue and uses this prediction to dynamically adapt the dialogue model of the system taking into consideration the user's needs and preferences. We have evaluated our proposal to develop a user-adapted spoken dialogue system that facilitates tourist information and services and provide a detailed discussion of the positive influence of our proposal in the success of the interaction, the information and services provided, and the quality perceived by the users.

## 1. Introduction

Dialogue systems are artificial systems able to hold a conversation with a human user, usually to achieve a certain objective (e.g., providing some information or fulfilling a task) through a natural language dialogue [1, 2]. With a spoken interface, the user is not restricted to traditional interfaces and so the number of contexts in which the system can be used grows, for example, with robots, with small devices, in environments where the user's eyes are busy (e.g., in-car systems), or with older adults or the disabled.

Such dialogues require a sequence of user and system turns until the objective is fulfilled. To organize these exchanges and gradually attain the user's objective, the system must be endowed with complex mechanisms to compute what the best system intervention is given the user's responses up to the current moment of the dialogue, to take the initiative in the conversation when necessary to lead the dialogue and maintain it within the limits of the system's domain of expertise, to request the information missing to achieve the objective, and to solve the possible miscommunication problems that may arise, including asking the user for clarifications.

Although it has been underlined that considering user models is of great importance to enhance the system services, these are usually not considered when designing the dialogue model for conversational agents, which center in solving the previously mentioned challenges for a general user [3, 4].

Adapting a conversational system to the peculiarities of its different users requires learning a user model jointly to the dialogue management process design. The application of statistical approaches to perform both tasks allows using real dialogues to explore a wider space of models (both user and dialogue models) if compared with rule-based systems [5, 6]. Although the definition of the parameters of a statistical model depends on expert knowledge about the application domain, the final objective is to develop more portable, robust, and adaptive conversational systems.

In this paper we present a framework to develop spoken dialogue systems able to adapt dynamically to the requisites and preferences of the user. The user model proposed estimates the user intention during the dialogue, that is, the information that the user is going to provide during the dialogue in order to achieve their objective. The prediction is performed after each system intervention, so that the adaptation can be achieved dynamically. To complete the adaptation a dialogue manager has been designed following a statistical approach based on neural networks that incorporates the user information generated by the user model along with the history of the dialogue.

Both models can be computed from a dialogue corpus using a format defined to code the information in a data structure that considers only whether each piece of information has been provided or not by the user and with which confidence it has been recognized by the speech recognition and understanding modules. This allows the proposed framework to be used in complex domains as it reduces the search space without losing relevant information.

We also describe an implementation of our proposal for the development of a spoken dialogue system information that provides user-adapted tourist information. We have evaluated the developed system and assessed the influence of the adaptation in the quality of the acquired dialogues and the information provided. The evaluation results show that the users perceive a higher quality and performance in the interaction with the system when our proposal is integrated.

The current paper presents the following important contributions. One of the main ideas of our proposal is that the dialogue system and the user model interact simultaneously, not offline as in our previous works [7, 8], so that the user model is employed in real time by the dialogue manager to decide the best answer dynamically and not in a posteriori laboratory evaluations. In addition, the technology used to build the proposed user model does not replicate that of the dialogue manager. This avoids the dialogues generated to be biased.

The novel user model incorporates numerous information sources to decide the next user action and integrates and orchestrates these heterogeneous sources and uses them significantly to make decisions. To optimize these computations it is very important to estimate the task underlying the current dialogue. Determining the task is one of the main innovations of the paper as it is used not only for the user simulator but also for the practical implementation of the dialogue system. Another important contribution is that such implementation is performed with a neural-network-based classifier trained for each of the tasks considered, so that a better selection of the next system can be attained weighting the outputs of these specialized classifiers.

All these decisions have been carefully designed so that the proposal can be portable across domains and applied to systems with varying complexity. In particular, the current paper shows the application of our proposal to develop a dialogue system that facilitates touristic information. This provides the benefit of showing an application of our scientific proposals in a task designed for a real system that provides a real service to real users. Finally, the overall evaluation of the proposal not only employs measures to compare the answers provided by the manager but also incorporates the evaluation of the system using objective and subjective measures obtained with real users.

The remainder of the paper is as follows. In Section 2 we describe the context and main motivation of our proposal and review main approaches focused on key aspects related to it, such as user modeling techniques when interacting with spoken conversational agents and the application of statistical methodologies for interaction management.  Section 3 presents in detail our proposal to develop user-adapted spoken conversational agents.  Section 4 shows a practical implementation of our proposal to develop a system providing tourist information and services.  Section 5 describes the evaluation results obtained after a comparative evaluation of a baseline version of this system with a user-adapted version that integrates our proposal. Finally, in Section 6 we describe some conclusions and guidelines for future work.

## 2. Related Work

Usually dialogue models are aimed at a generality of users and are not developed to consider their peculiarities through specific user models [9–11]. However, some authors have addressed before the challenge of building adaptive systems through different adaptation levels [12]. A simple approach is to use different user profiles containing the preferences of individual users or user groups, which the users have expressed prior to using the system; this is frequently used in ambient intelligence environments where the system considers the user preferences to operate the environment.

Other approaches attempt to adapt to the users dynamically by detecting their needs instead of explicitly asking the users to introduce the parameters. This has been used, for example, to adapt to the users by detecting their skills while using the system [13–15] and also to detect the variable intentions of the user during the dialogue and how these are affected by the system interventions [16, 17].

Modeling the dialogue using intentions allows overcoming of the ambiguity introduced by natural language when the communication process considers the words uttered [18], as dialogue acts can be used to describe and represent intentions [19, 20]. Dialogue acts have been used to represent key aspects in dialogue systems, from corpus annotation to the design of dialogue models or the representation of the behavior of interlocutors during the conversation. The semantic representation of information can also be achieved through other models such as Hybrid Deep Belief Networks (HDBNs) [21], time-dependent semantic similarities [22], fuzzy set theories [20], or ontologies [23].

The use of *n*-grams to predict the user intention was introduced in [24]. The model predicts the most probable action according to the actions defined for the user and the previous dialogue history. As *n*-grams limit the dialogue history to the previous *n* user actions (usually 2), sometimes important information is lost and the response selected by the model makes sense in the context of the previous user intervention but not in the whole dialogue.

In [17] a user model is proposed based on Hidden Markov Models to represent the previous states and history of the dialogue in more detail. The proposed model is based on the theory of Information States [16], feature vectors that include the current state, the previous history of the dialogue, and the possible user actions. In [25] an extension is proposed to represent both the user and the system behavior by training a specific model for each of the objectives of the dialogue.

In [26] a method is proposed to simulate the user intention by integrating different levels of discourse knowledge with Markov models and regression. In [27] the model proposed also incorporates acoustic features to increase the accuracy of the intention prediction, which are extracted during the speech recognition process from the words uttered and the confidence measures computed by the recognizer.

The approach described in [28] uses other features for the prediction of the user intention, a mixture of morpheme, discourse, and domain level characteristics that are integrated by means of a maximum entropy model. In [29] a technique is proposed that uses information about the activities performed by the users as a solution to disambiguate their inputs. This information is achieved with a reinforcement learning algorithm and then used by the dialogue manager to decide the next system action.

The user simulator technique in [30] is based on an agenda that stores the possible objectives of the user during the dialogue and represents the interaction as a sequence of states and dialogue acts that define the user plan and that may vary during the dialogue. This structure is implemented as a stack that stores the dialogue acts which are pending in order for the user to provide all the information required to achieve their objective within the dialogue. The agenda and the objective are updated dynamically during the conversation.

As described in Section 3, the technique that we propose to model the user intention combines different information sources and heuristics to enhance the dialogue model. It can easily be integrated with the dialogue manager and can be used to automatically simulate dialogues by means of its interaction with the system, thus decreasing the effort necessary to enhance the dialogue model.

Once the user model has been learnt, it is necessary to define how it is going to be used by the dialogue manager to adapt the system. In [31–33] the main architectures and methodologies for dialogue management are described. The use of statistical techniques to automatize the process reduces the time and effort needed for the development of new managers and allows covering new requisites and makes the system portable to new tasks and users [9], then increasing the system scalability.

The main statistical methodology for dialogue management relies on decision Markov models and reinforcement learning to model the dialogue as an optimization problem [34, 35]. One of the main drawbacks of this proposal is the difficulty to apply it to real tasks with a high number of states [36]. Partially Observable Markov Models (POMDPs), though also limited in real tasks [36], offer a possibility to explicitly represent uncertainty in human-machine communication [37]. Other statistical proposals for dialogue management combine POMDPs with rule-based methodologies [18, 38] or create partitions in the search space to make it more tractable [39, 40]. Also they have been merged with Bayesian models to favour learning the best features [41, 42]. Other recent works are merged with Hidden Markov Models [25] and Finite-State Transducers [43, 44].

The statistical methodology that we propose for dialogue management is based on using a data corpus to learn the next system action considering the history of the dialogue up to the current moment and the user model. This is a benefit of our proposal with respect to the state of the art, enhanced by the fact that the data structure used makes it possible to store the information efficiently to adapt to different tasks.

In our previous work we have evaluated different classifiers widely used in the field of natural language processing and speech technologies [6–8]. Neural networks have outperformed the other classifiers in different application domains [6, 45–48].

## 3. Proposed Framework to Develop User-Adapted Spoken Dialogue Systems

**Figure 1:**
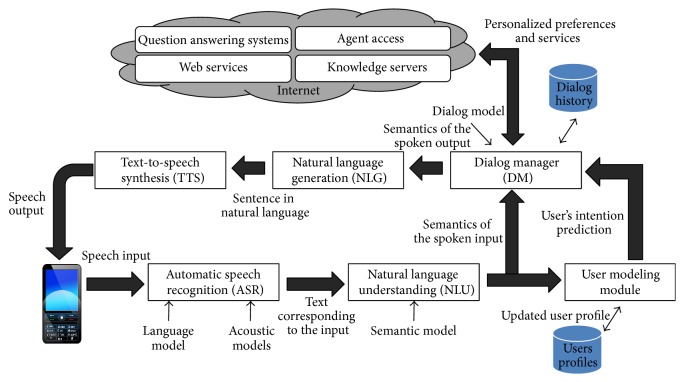
Architecture to develop user-adapted spoken conversational agents.

Figure 1 shows the architecture that integrates our proposed framework to generate adaptive spoken conversational agents. A user modeling module considers the previous dialogue interactions and specific features of the user (defined by means of user profiles) to predict the user intention, defined as the next user action, which we represent by one or more dialogue acts as described in the previous section.

The dialogue manager takes as input this prediction, the current user utterance, and the sequence of user and system dialogue acts until the current moment. Using this information it selects the next system action (next system dialogue act). The following subsections describe the statistical methodologies proposed for the development of the two modules.

### 3.1. User Modeling

Our proposed technique for user modeling simulates the user intention providing the next user dialogue act in the same representation defined for the spoken language understanding module. If we denote the user and system interventions at a certain moment *i* as *U*_*i*_ and *A*_*i*_, respectively, a dialogue can be modeled as a sequence of pairs (*A*_*i*_, *U*_*i*_) where *A*_0_ is the initial system prompt and *U*_*n*_ is the system response to the system prompt *A*_*n*_. Each of these interventions is modeled as dialogue acts.

The lexical, syntactic, and semantic information associated with the speaker *u*'s *i*th turn (*U*_*i*_) is denoted as *c*_*i*_^*u*^. This information is usually represented by the following:

- the words uttered;
- part of speech tags, also called word classes or lexical categories; common linguistic categories include noun, adjective, and verb;
- predicate-argument structures, used by SLU modules in various contexts to represent relations within a sentence structure; they are usually represented as triples (subject-verb-object);
- named entities or groups of words that identify objects, for example, proper names, dates, and numerical expressions.

The model proposed to simulate the user intention is based on enhanced version of the one presented in [49]. Each user action is represented by means of the subtask in the dialogue where it belongs, the named entities spotted, and the dialogue acts that intervene in the user utterance. For instance, for the user action corresponding to conveying the origin of a trip, the subtask may be timetable query, the named entity could be the name of the origin city, and the dialogue act could be coded as* Provide-Origin*.

For speaker *u*, DA_*i*_^*u*^ denotes the dialogue label of the *i*th turn, and ST_*i*_^*u*^ denotes the subtask label to which the *i*th turn contributes. The next dialogue act in the user model is computed as indicated in (1) from the *k* previous utterances (dialogue history) and the lexical, syntactic, and semantic features extracted from the words uttered by the user:

(1) $$\begin{array}{c} DA_{i}^{u}=\underset{d^{u}\in D}{\mathrm{argmax}}\;P\left(\;d^{u}\;|\;c_{i}^{u},ST_{i-1}^{i-k},DA_{i-1}^{i-k},c_{i-1}^{i-k}\;\right). \end{array}$$

The dialogue act computed is then used together with the dialogue history (*k* previous interventions) and the characteristics of the user utterance to determine the subtask to which the user intervention contributes as indicated in the following:

(2) $$\begin{array}{c} ST_{i}^{u}=\underset{s^{u}\in S}{\mathrm{argmax}}\;P\left(\;s^{u}\;|\;DA_{i}^{u},c_{i}^{u},ST_{i-1}^{i-k},DA_{i-1}^{i-k},c_{i-1}^{i-k}\;\right). \end{array}$$

In our proposal, we consider static and dynamic features to estimate the conditional distributions shown in (1) and (2). Dynamic features include the dialogue act and the task/subtask. Static features include the words in each utterance, the dialogue acts in each utterance, predicate-arguments in each utterance, and also a set of features included in a user profile. All pieces of information are computed from corpora using *n*-grams, that is, computing the frequency of the combination of the *n* previous words, dialogue acts, or predicate-arguments in the user turn.

The user profile includes a unique identifier for the user, their expertise level, gender, most frequent queries, and the device that they usually employ to communicate with the system.

The previous equations are solved in [49, 50] using the maximum entropy technique, which allows obtaining the average parameters from a corpus. Equation (3) shows how this estimation can be computed by means of a Gibbs distribution of weighted (*λ*) parameters, where *X* refers to the distributions in (1) and (2) (DA_*i*_^*u*^ or ST_*i*_^*u*^) and *ϕ* corresponds to our feature vector:

(3) $$\begin{array}{c} P\left(\;X=st_{i}\;|\;\phi \;\right)=\frac{e^{\lambda_{st_{i}}\cdot \phi }}{\sum_{st=1}^{V}e^{\lambda_{st_{i}}\cdot \phi }}. \end{array}$$

This calculation fastens the calculation of the distributions, which in turn allows using bigger corpora if compared to state of the art approaches [49–51]. The binary classifiers for each task are computed using

(4) $$\begin{array}{c} P\left(\;y\;|\;\phi \;\right)=1-P\left(\;\bar{y}\;|\;\phi \;\right)=\frac{e^{\lambda_{y}\cdot \phi }}{e^{\lambda_{y}\cdot \phi }+e^{\lambda_{\bar{y}}\cdot \phi }}=\frac{1}{1+e^{-\lambda_{\bar{y}}^{\prime }\cdot \phi }}, \end{array}$$

where $\lambda_{\bar{y}}$ is the parameter vector for the antilabel $\bar{y}$ and $\lambda_{\bar{y}}^{\prime }=\lambda_{y}-\lambda_{\bar{y}}$.

### 3.2. Interaction Management

In the dialogue management process a dialogue sequence *S*_*i*_ represents the pair (*A*_*i*_, *U*_*i*_). The aim is to select the best system response according to the dialogue sequences up to the current moment. This is processed as shown in the following:

(5) $$\begin{array}{c} {\hat{A}}_{i}=\underset{A_{i}\in A}{\mathrm{argmax}}\;P\left(\;A_{i}\;|\;S_{1},\ldots ,S_{i-1}\;\right), \end{array}$$

where set *𝒜* contains all the possible system answers.

The main challenge for this calculation is the high number of dialogue sequences that it may have to deal with, even in simple application domains. We tackle this problem using a data structure, the interaction register (IR), in which we store the information provided by the user during the dialogue in terms of dialogue acts, the act prediction generated by (1), and the subtask prediction provided by (2).

At each moment *i*, the IR contains an abstract representation of the information that has been provided independently of the order in which it has been communicated. This allows reducing the number of possible dialogue histories to consider, so that the selection of the system response can be computed even in complex tasks as shown in (6). As can be observed, the previous dialogue sequence (*S*_*i*−1_) is also considered in the calculation. This is due to the task-independent dialogue acts, such as confirmations or negations, that demand considering the previous turn:

(6) $$\begin{array}{c} {\hat{A}}_{i}=\underset{A_{i}\in A}{\mathrm{argmax}}\;P\left(\;A_{i}\;|\;IR_{i-1},S_{i-1}\;\right). \end{array}$$

The IR only considers the dialogue acts and not the actual values provided. This way, each position of the IR can only take one out of three possible values: 0 when the user has not conveyed the information, 1 when it has been conveyed and processed with high confidence [52], or 2 when it has been conveyed but the recognition confidence is low and thus it probably requires confirmation. As it has been previously described, (6) is solved by means of a neural network whose input is the pair (IR_*i*−1_, *A*_*i*_) and outputs the probability of selecting each of the possible system answers.

**Figure 2:**
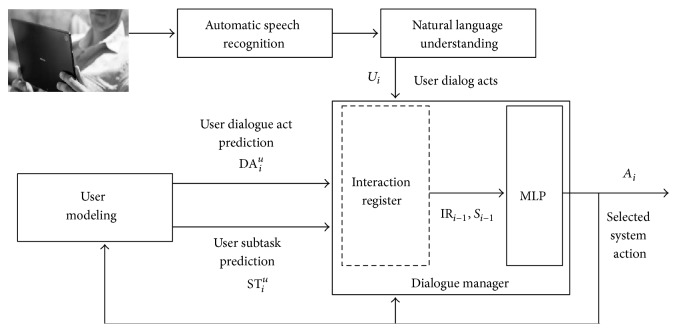
Integration of the proposed user model and dialogue management methodology for the development of user-adapted spoken dialogue agents.

Figure 2 summarizes the combination of the proposed user modeling and dialogue management methodologies. As can be observed, the user modeling module provides a prediction of the next user dialogue act and the current subtask of the dialogue. The set of user dialogue acts and predicted values for the current user's dialogue act and subtask are used to update the interaction register. The dialogue manager considers this register and the last system response for the selection of the next system action.

### 3.3. MLP Classifier

The codification of the input pair (IR_*i*−1_, *S*_*i*−1_) for the MLP includes the following variables:

(i) The last system response (*A*_*i*−1_) is a variable with the same number of bits compared to possible system responses (*C*)
  (7) $$\begin{array}{c} {\vec{x}}_{1}=\left(\;x_{1_{1}},x_{1_{2}},x_{1_{3}},\ldots ,x_{1_{C}}\;\right)\in {\left\{\;0,1\;\right\}}^{C}. \end{array}$$
(ii) The interaction register (IR_*i*−1_) is modeled by the variables required to model each of the user dialogue acts that are dependent on the task (each one modeled using a variable with three bits, which are related to the previously described {0,1, 2} values), the predicted current user dialogue act obtained with (1) (variable with the same number of bits compared to possible user responses, *N*), and the predicted dialogue subtask obtained with (1) (variable with the same number of bits compared to possible subtasks, *T*):
  (8) x→i=xi1,xi2,xi3∈0,13i=2,…,N+1,x→N+2=x11,x12,x13,…,x1N∈0,1N,x→N+3=x11,x12,x13,…,x1T∈0,1T.
(iii) The task-independent information is modeled using three variables, which are, respectively, related to the dialogue acts used by the user to provide an affirmation or a rejection or to indicate that they have not understood the previous system's action. Each one of these dialogue acts is modeled using a variable with the same number of bits compared to possible values ({0,1, 2}). Consider
  (9) $$\begin{array}{c} {\vec{x}}_{i}=\left(\;x_{i_{1}},x_{i_{2}},x_{i_{3}}\;\right)\in {\left\{\;0,1\;\right\}}^{3}\;i=N+4,\ldots ,N+6. \end{array}$$

## 4. Case Application: Development of a Dialogue System Providing Tourist Information and Services

We have developed a tourist information dialogue system that integrates our proposal for user adaptation. The system provides information about navigation, hotels, interesting spots and monuments, restaurants and bars, shopping, cultural and sport activities, and public transportation taking into account the preferences and physical situation of the user in a city. The information provided is retrieved from different web repositories, most of them updated daily.

**Figure 3:**
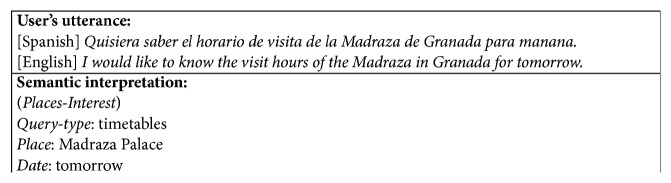
An example of the labeling of a user turn in the tourist information system.

We have defined 10 types of queries with 115 user dialogue acts corresponding to the pieces of data that the user must provide (*Places-Interest*,* Weather-Forecast*,* Hotel-Booking*,* Restaurants-Bars*,* Shopping*,* Street-how-to-get*,* Cultural*,* Sport*,* Festivities*, and* Public-Transport*) and 3 task-independent dialogue acts (*Affirmation*,* Negation*, and* Not-Understood*). The IR defined contains 129 fields corresponding to the dialogue acts described.  Figure 3 illustrates how a user utterance can be represented using our proposal.

## 5. Evaluation of Our Proposal for the Tourist Information System

We have evaluated the system with respect to a baseline that employs the same approach for dialogue management but does not incorporate the user model [53].

To assess the benefits of our proposal to include user adaptation, we have evaluated the developed system for the specific scenario of the city of Granada (Spain) and compared it to the baseline for the same city. In order to do so, 150 recruited users have followed a set of scenarios that specifies a set of objectives that must be fulfilled by the user at the end of the dialogue and are designed to include the complete set of functionalities previously described for the system. The number of recorded dialogues was 600: 300 dialogues were acquired by 75 users using the baseline version of the system and 300 dialogues were acquired by 75 users using the user-adapted system. An example of the defined scenarios is as follows:

 
User name: Jose Madrigal
 
Location: Periodistas Street
 
Date and Time: 2015-04-03, 19:45
 
Device: MOT-TVS-2-GP 01-00-34-7B-06-A7
 
Objective: Cultural activities for today.
 
Listings for next weekend

**Figure 4:**
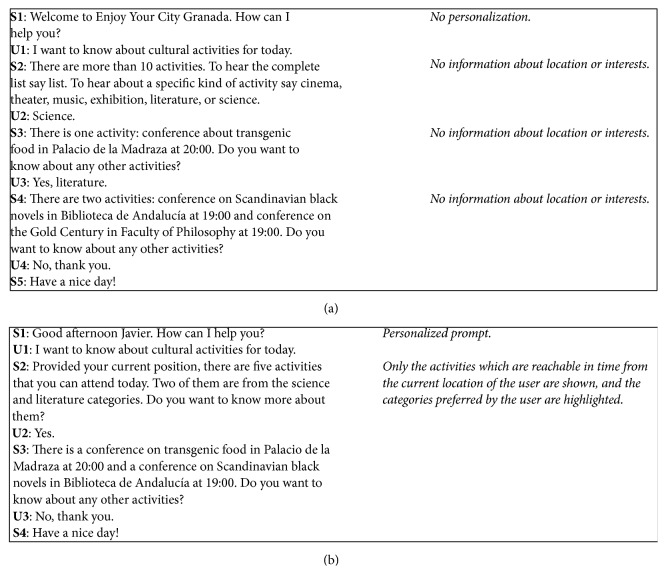
An example of a dialogue for the tourist domain using the initial system (a) or the user-adapted system (b).

For illustrative purposes, Figure 4 shows a dialogue corresponding to the previous scenario acquired with the baseline and user-adapted systems (U: user turns; S: system turns). As can be observed, the user-adapted system shows a tendency of providing the required services with higher agility and using more natural answers than the baseline.

We have carried out an objective and subjective assessment to compare the two versions of the system. The objective evaluation was aimed mainly at detecting the nature of the information exchanged and the quality and duration of the exchanges: dialogue success, error correction, dialogue and turn duration, frequency of dialogues and number of different dialogues generated, and proportion of user versus system actions during the dialogue. The measures defined to complete this evaluation are summarized as follows [54, 55].

*Evaluation Measures Based on the Interaction Parameters Gathered from the Dialogues Acquired with the Two Versions of the Enjoy Your City System*

*Dialogue Success*

 Dialogue success rate (%success): the percentage of successfully completed tasks. In each scenario, the user has to obtain one or several pieces of information, and the dialogue success depends on whether the system provides the correct data (according to the aims of the scenario) or incorrect data to the user.
 Average number of corrected errors per dialogue (nCE).
 The average of errors detected and corrected by the dialogue manager. We have considered only the errors that modify the values of the attributes and that could cause dialogue failure.
 Average number of uncorrected errors per dialogue (nNCE): the average of errors not corrected by the dialogue manager. Again, only errors that modify the values of the attributes are considered.
 Error correction rate (%ECR) which is the percentage of corrected errors, computed as nCE/(nCE + nNCE).

*High-Level Dialogue Features*

 Average number of turns per dialogue (avgturns/dial).
 Percentage of different dialogues (%diff).
 Number of repetitions of the most seen dialogue (#repMS).
 Number of turns of the most seen dialogue (#turnsMS).
 Number of turns of the shortest dialogue (#turnsSh).
 Number of turns of the longest dialogue (#turnsLo).
 Ratio users versus system actions (us/sysAct).

*Dialogue Style/Cooperativeness Measures*

 System dialogue acts: confirmation of concepts and attributes, questions to require information, and answers generated after a database query.
 Confirmation rate (%confirm) which was computed as the ratio between the number of explicit confirmations turns (nCT) and the number of turns in the dialogue (nCT/nT).
 User dialogue acts: request to the system, providing information, confirmation, yes/no answers, and other answers.
 Goal-directed actions versus grounding actions: goal-directed actions are requesting and providing information, while grounding actions are explicit and implicit confirmations, dialogue formalities (greetings, instructions, etc.), and unrecognized actions.

We then used two-tailed *t*-tests to compare the means across the different types of scenarios and users as described in [54]. The significance of the results was computed using the SPSS software [56] with a significance level of 95%. (The degrees of freedom employed for *t*-tests are *N* − 1 in case the compared groups have the same number of samples (*N*) and *N*1 + *N*2 − 1 when they differ in the number of samples (*N*1 and *N*2).)

To account for the users' perception, we conducted a subjective evaluation by means of the following questionnaire with responses in a 5-point Likert scale: (Q1) the system understood me; (Q2) I understood the system messages; (Q3) it was easy to obtain the information I was looking for; (Q4) the interaction pace was adequate; (Q5) if the system made recognition mistakes, I could solve them easily. The answers for the different questions were the same: never/not at all, seldom/in some measure, sometimes/acceptably, usually/well, and always/very well.

The objective evaluation was aimed mainly at detecting the nature of the information exchanged and the quality and duration of the exchanges: dialogue success, error correction, dialogue and turn duration, frequency of dialogues and number of different dialogues generated, and proportion of user versus system actions during the dialogue.

Table 1 shows the results; as can be observed both systems attained good success rates, though the system with user model outperforms the baseline (85% versus 97%). The user-adapted dialogue obtained a better user error detection and correction rate, though this improvement was not statistically significant using a *t*-test. This may be because we did not incorporate any improvement on the recognition and understanding modules with respect to the baseline. Though this was not an objective for incorporating the user model, it makes it possible to correct errors faster when they are detected during the dialogue.

Also with the adapted system the duration of the successful dialogues was shorter (8.1 turns in average instead of the average 12.2 turns of the baseline), as the users are less often required to provide additional information because the system is already adapted to them. This reduction showed to be significant with the *t*-test. The adaptation also makes it possible for the users to choose between a wider range of alternatives, as the number of turns of successful dialogues presents a higher deviation than in the baseline. Also the number of user turns is higher than the number of system turns, and the nature of such turns is more in providing new values than in confirming information that was already provided.

Regarding the dialogue participant activity, the dialogues acquired with the user-adapted version of the system have a higher proportion of system actions, as less information requires confirmation in the user-adapted system. There is also a slight reduction in the mean values of the turn length; these dialogues are statistically shorter, as they provide 1.41 actions per user turn instead of the 1.67 actions provided by the baseline dialogues. This is again because the users have to explicitly provide and confirm more information in the baseline system. The results of the *t*-test in a comparative analysis of this measure showed a significant difference (significance value of 0.029).

Regarding the measures used to evaluate the style of the dialogues, we counted the number of each of the possible actions of the system and the user. To do this, the system's actions have been grouped into confirmations of queries and user-supplied values (*S*_*confirm*), questions to prompt the user (*S*_*request*), and system responses generated after consultation system information repositories (*S*_*inform*). User actions have been grouped into queries to the system (*U*_*query*), actions in which the user provides information required by the system (*U*_*provide*), confirmation of queries and values previously provided (*U*_*confirm*), affirmations and rejections (*U*_*yesno*), and other actions not included in the previously described categories (*U*_*other*).

Table 2 shows that users need to provide less information using the user-adapted system. This explains the higher proportion for the rest of user actions with regard to the baseline system (both differences significant over 98%). There is also a higher proportion of yes/no actions for the user-adapted dialogues, which was not significant in the *t*-test.  Table 3 shows a higher proportion of the inform and confirmation system actions when this system is used in comparison with the baseline system (both differences significant over 98%). A higher proportion of yes/no actions for the user-adapted dialogues can also be observed. These actions are mainly used to confirm that the specific queries have been correctly provided.

We also measured the ratio of actions to achieve the objective(s) of the dialogue (actions in which queries are performed and/or information is provided), actions that increase the number of turns (especially confirmations and rejections), and remaining actions.  Table 4 shows a comparison between these categories. As can be observed, the dialogues provided by the user-adapted system have a better quality, as the proportion of goal-directed actions is higher. This difference showed a significance value of 0.029 in the two-tailed *t*-test.

Finally, Table 5 shows the results of the subjective evaluation. The values obtained show that both systems were successful, though the adapted system was perceived as having a more appropriate pace, as it is faster, and thus that it was easier to find the information required.

## 6. Conclusions and Future Work

In this paper, we present a framework to build user adaptive dialogue systems based on statistical dialogue management enhanced with a dynamically computed user model. In our proposal, the user model computes the most probable next user turn and merges it with information about the user preferences and needs, so that it is considered by the dialogue manager along with the complete history of the interaction in order to adapt the interaction. These calculations are performed automatically using an abstract representation of the interaction structure based on dialogue acts that simplifies the search space and makes our proposal appropriate even for complex interactions and portable across domains.

To show the benefits of our proposal we have implemented a dialogue system that provides touristic information employing the framework and compared it with a baseline system that uses the same dialogue management process but does not incorporate the user model. In order to perform the evaluation, we have performed a series of experiments with recruited users recording the interaction parameters as well as assessing their subjective opinion.

The results show that the system that incorporates our framework obtained a higher success rate in the provision of the adapted services. By means of the user-adapted system, the time required to provide the information can be reduced. In addition, the interaction was more fluid and the dialogues with the adapted system present a better ratio of goal-directed actions selected by the system to successfully provide the different services, thus decreasing the actions that might discourage users (e.g., confirmations or re-request of information).

For future work we plan to build other systems in different application domains in order to demonstrate empirically that our proposal can be used with different user and dialogue models of varying complexity. We also plan to extend our proposal to dialogues that cannot be translated into a form-filling structure, such as companions or health coaches.

## Figures and Tables

**Table 1:** Results of the high-level dialogue features defined for the comparison of the baseline and user-adapted systems.

	Baseline system	User-adapted system
%success	85%	97%
nCE	0.83	0.90
nNCE	0.18	0.09
%ECR	81%	92%
avgturn/dial	12.2	8.1
%diff	85%	83%
#repMS	5	8
#turnsMS	9	7
#turnsSh	7	7
#turnsLo	17	15

**Table 2:** Distribution of the user dialogue acts in both systems.

	Baseline system	User-adapted system
*U*_*query*	31.74%	33.43%
*U*_*provide*	21.72%	19.98%
*U*_*confirm*	10.81%	9.34%
*U*_*yesno*	33.47%	35.77%
*U*_*other*	2.26%	1.48%

**Table 3:** Distribution of the system dialogue acts in both systems.

	Baseline system	User-adapted system
*S*_*confirm*	13.31%	11.16%
*S*_*request*	18.14%	16.27%
*S*_*inform*	67.63%	71.84%
*S*_*other*	0.92%	0.73%

**Table 4:** Dialogue spent on goal-directed actions, ground actions, and other possible actions.

	Baseline system	User-adapted system
Goal-directed actions	66.37%	74.16%
Grounding actions	32.34%	24.39%
Rest of actions	1.29%	1.45%

**Table 5:** Results of the subjective evaluation of the baseline and user-adapted systems with real users (1 = worst and 5 = best evaluation).

	Baseline system	User-adapted system
Q1	M = 4.61, SD = 0.37	M = 4.83, SD = 0.34
Q2	M = 3.63, SD = 0.24	M = 3.97, SD = 0.26
Q3	M = 3.84, SD = 0.56	M = 4.35, SD = 0.35
Q4	M = 3.41, SD = 0.28	M = 4.24, SD = 0.29
Q5	M = 3.25, SD = 0.59	M = 3.36, SD = 0.57
